# AI-Enabled Algorithm for Automatic Classification of Sleep Disorders Based on Single-Lead Electrocardiogram

**DOI:** 10.3390/diagnostics11112054

**Published:** 2021-11-05

**Authors:** Erdenebayar Urtnasan, Eun Yeon Joo, Kyu Hee Lee

**Affiliations:** 1 Artificial Intelligence Bigdata Medical Center, Wonju College of Medicine, Yonsei University, Wonju 26417, Korea; edenbyra@yonsei.ac.kr; 2 Samsung Medical Center, Department of Neurology, School of Medicine, Sungkyunkwan University, Suwon 16419, Korea; eunyeon.joo@gmail.com

**Keywords:** sleep disorders, automatic classification, electrocardiogram, deep learning, convolutional neural network

## Abstract

Healthy sleep is an essential physiological process for every individual to live a healthy life. Many sleep disorders both destroy the quality and decrease the duration of sleep. Thus, a convenient and accurate detection or classification method is important for screening and identifying sleep disorders. In this study, we proposed an AI-enabled algorithm for the automatic classification of sleep disorders based on a single-lead electrocardiogram (ECG). An AI-enabled algorithm—named a sleep disorder network (SDN)—was designed for automatic classification of four major sleep disorders, namely insomnia (INS), periodic leg movement (PLM), REM sleep behavior disorder (RBD), and nocturnal frontal-lobe epilepsy (NFE). The SDN was constructed using deep convolutional neural networks that can extract and analyze the complex and cyclic rhythm of sleep disorders that affect ECG patterns. The SDN consists of five layers, a 1D convolutional layer, and is optimized via dropout and batch normalization. The single-lead ECG signal was extracted from the 35 subjects with the control (CNT) and the four sleep disorder groups (seven subjects of each group) in the CAP Sleep Database. The ECG signal was pre-processed, segmented at 30 s intervals, and divided into the training, validation, and test sets consisting of 74,135, 18,534, and 23,168 segments, respectively. The constructed SDN was trained and evaluated using the CAP Sleep Database, which contains not only data on sleep disorders, but also data of the control group. The proposed SDN algorithm for the automatic classification of sleep disorders based on a single-lead ECG showed very high performances. We achieved F1 scores of 99.0%, 97.0%, 97.0%, 95.0%, and 98.0% for the CNT, INS, PLM, RBD, and NFE groups, respectively. We proposed an AI-enabled method for the automatic classification of sleep disorders based on a single-lead ECG signal. In addition, it represents the possibility of the sleep disorder classification using ECG only. The SDN can be a useful tool or an alternative screening method based on single-lead ECGs for sleep monitoring and screening.

## 1. Introduction

Sleep is an essential physiological need for everyone, and it can refresh and restore the human body. In addition, both the quality and quantity of sleep are very important to live a healthy life [1,2]. However, many types of sleep disorders such as sleep apnea [3], insomnia (INS) [4], periodic leg movement (PLM) [5], and REM sleep behavior disorder (RBD) [6], can destroy sleep quality. Sleep disorders can lead to an increased risk of occurrence of a number of negative health conditions, including daytime sleepiness [7], headache [8], cardiovascular disease [9], decreased cognitive function [10], and decreased immunity [11]. The number of people who suffer from sleep disorders (e.g., insomnia, sleep fragmentation, and sleep apnea) is increasing; thus, it is necessary to diagnose these problems correctly through systematic sleep monitoring [12].

Nocturnal polysomnography (PSG) is a gold-standard diagnostic tool for sleep disorders, including INS, PLM, RBD, and nocturnal frontal-lobe epilepsy (NFE). To evaluate sleep disorders, subjects go to sleep at a sleep center, and sensors are attached to the body for measuring physiological signals (electroencephalogram (EEG), electrooculography, electromyography (EMG), etc.) [13]. Based on the physiological signals acquired via PSG for the patient, expert or licensed sleep technicians can objectively diagnose sleep disorders. However, the use of PSG has some limitations such as high cost and inconvenience (e.g., time consumption for applying multiple sensor attachments). In addition, the annotation and labeling of PSG recordings (presenting numerous results) by sleep technicians are laborious tasks.

Electrocardiography (ECG) is a physiological signal that can represent cardiopulmonary activity [14]. In addition, ECG is regarded as an alternative physiological source for digital healthcare and digital medicine as it has the most informative signal that contains not only cardiac activity (as beat-to-beat interval, heart rate, and QRS complex) but also respiratory activity including ECG-derived respiratory activity [15]. There are many clinical pieces of evidence and studies in which the relation between ECG and sleep disorders has been noticed [16,17]. In the early 2000s, various studies proposed alternative methods based on ECG analysis for digital healthcare and digital medicine [18]. Some studies have used ECG to automatically detect sleep disorders including sleep apnea [19,20], sleep stages [21,22,23,24,25,26,27], periodic leg movement [28], and insomnia [29]. These studies used various machine learning and deep learning methods such as the support vector machine (SVM), artificial neural network (ANN), and convolutional neural network (CNN). They proposed alternative detection methods for just one sleep disorder, from one or multiple input sources, based on machine learning. In addition, these studies used handcrafted feature sets extracted using canonical machine-learning methods. However, a multiclass classification-based study should be required that can be automatically classifying sleep disorders such as INS, PLM, RBD, and NFE from an ECG signal for more convenient digital healthcare services.

Rapidly advancing technologies, including big data, artificial intelligence (AI), the Internet of Things, and cloud computing, are changing the medical trend from conventional healthcare to digital medicine and health. Currently, there are approximately thirty AI-based medical devices approved by the FDA as software as a device services for digital healthcare and digital medicine [30]. A few of them were related to the analysis of cardiac activity studies, including the echocardiogram [31], ECG analysis [32], and cardiac monitor system [33], to support the clinical decisions of physicians. The AI-based sleep-scoring solution is called EnsoSleep [34], but it is targeted at sleep centers or hospitals to support the automatic annotation for sleep technicians. Therefore, a convenient, accurate, and automatic method to the classification of sleep disorders for everyone in daily life is important not only clinically but economically and socially.

The proposed AI-enabled algorithm was designed by a sleep disorder network (SDN) based on deep learning for multiclass classification of sleep disorders using a single-lead ECG signal. The designed SDN was used to achieve multiclass classification for different sleep disorders, including INS, PLM, RBD, and NFE, and the control (CNT) group. We aimed to demonstrate novel features or specific patterns for each sleep disorder in the ECG signal in this study. Thus, the single-lead ECG signal was used without extracting any intermediate vital signs, including peak-to-peak interval, heart rate, and heart rate variability, and any other hand-crafted features.

## 2. Materials and Methods

**Figure 1:**
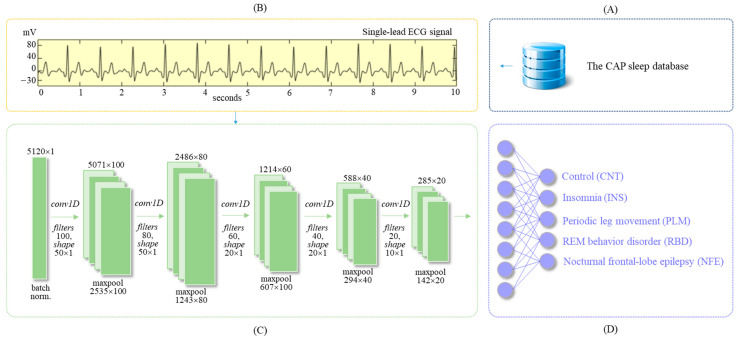
Schematic diagram of this study. (**A**) CAP sleep database, (**B**) ECG dataset, (**C**) deep learning model, and (**D**) Outputs.

This paper proposes an AI-enabled algorithm for automatic classification of sleep disorders, including INS, PLM, RBD, and NFE, based on a deep learning SDN model using a single-lead ECG signal. The proposed method consists of four main parts: The CAP sleep database (Figure 1A), ECG dataset (Figure 1B), deep learning model (Figure 1C), and outputs (Figure 1D). Each part is detailed in the following subsections.

### 2.1. The CAP Sleep Database

In this study, we used the cyclic alternative pattern (CAP) sleep database, including 108 PSG recordings, measured at the sleep disorders center of the Ospedale Maggiore of Parma, Italy [35]. It is a PSG study for the CAP in EEG activity occurring during non-rapid eye movement (NREM) sleep. The CAP sleep database covers not only the healthy (16) subjects but also the diverse sleep disorders, including NFE (40 subjects), RBD (22 subjects), PLM (10 subjects), INS (9 subjects), narcoleptic (5 subjects), sleep-disordered breathing (4 subjects), and bruxism (2 subjects). In addition, certified sleep experts at the sleep center annotated the scoring of the sleep structure according to the rules of Rechtschaffen and Kales [36].

A total of 35 subjects were enrolled from five different groups including the CNT, NFE, RBD, PLM, and INS for this study. A total of 7 subjects were randomly selected from each subject group to design the proposed SDN model for the automatic classification of sleep disorders (Table 1).

### 2.2. ECG Dataset

The ECG signal was extracted from the PSG recordings of the CAP Sleep Database. It has a sampling frequency of 512 Hz. Every recording is segmented at 10-s intervals, and each segment has 5120 samples. A total of 115,837 segments were obtained after combining the 35 subjects from the five different groups to create the entire ECG dataset. For the training and testing of the constructed SDN model, datasets were built from randomly selected subjects from each subject group (Table 2). The training set comprised 74,135 episodes from 28 subjects, whereas the test set comprised 23,168 episodes from 7 subjects (Figure 1B).

### 2.3. Deep Learning Algorithm

The proposed SDN model is a deep learning algorithm (Figure 1C) that was designed to discriminate the morphological characteristics and temporal patterns for each sleep disorders from a single-lead ECG signal. The SDN was implemented by CNN that can extract high-dimensional feature maps and patterns from the input ECG signal. Because the input ECG signal is a one-dimensional (Figure 1D) time series, 1D convolution, gated recurrent unit (GRU) [37], and 1D max pooling were used to construct the SDN for sleep disorder classification. In addition, the SDN was optimized to enable its application in clinical fields and sleep screening. For the optimization of the SDN, batch normalization [38], dropout [39], and a rectified linear unit (ReLU) [40] were appropriately set and used through trial and error. These techniques are detailed as follows.

Convolution layer: It is appropriate for application to physiological signals, such as an ECG, because it is simpler and faster than two-dimensional convolutions. The 1D convolution can be represented as

(1) $$x_{k}=b_{k}+\sum_{i=1}^{N}w_{k}\times y_{i}$$

where *x_k_* is the *k*-th feature map, *b_k_* is the bias of the *k*-th feature map, *w_k_* is the *k*-th convolutional kernel from all features of the *k*-th feature map, and *y_i_* represents the *i*-th feature map.

Pooling layer: Pooling can reduce the dimensions of the intermediate feature maps. If the 1D kernel is used in the pooling operation, it can be called 1D pooling. All the pooling layers use max pooling.

Gated recurrent unit: GRU is one of the improved architectures of the recurrent neural network that invented by K. Cho [37]. It has only two gates: an update gate, *z*, and a reset gate, *r*. The reset gates can capture short-term dependencies, whereas update gates help to capture long-term dependencies in the input ECG signals.

Batch normalization: Before training, the generated SDN model, batch normalization is applied to the input ECG signal, as presented in Equation (2).

(2) $$x_{b}=\alpha \times \left(\frac{x_{i}-\mu }{\sqrt{\sigma^{2}+\varepsilon }}\right)+\beta$$

where *ε* is a small random noise, *μ* is the mini-batch mean, *σ* is the mini-batch variance, *α* is a scale parameter, and *β* is a shift parameter. Both *α* and β are trainable and updated in an epochwise manner.

Dropout: In this technique, random nodes in a network are dropped out to reduce overfitting in the network model by preventing complex adaptations on training data.

ReLU: It was used as the activation function of each layer of the SDN, and it can be represented as

(3) f(x)=max(0,wx+b)

where *x* is the feature map, *w* is the weight, and b is the bias. ReLU delivered robust training performance and consistent gradients, thereby facilitating gradient-based learning [40].

Table 3 presents the detailed structure and characteristics of the final architecture of the constructed SDN; the algorithm consists of five-layer 1D convolution with 1D max pooling and dropout. In each convolutional layers (100, 80, 60, and 40), the kernel sizes were 50 × 1, 40 × 1, 30 × 1, and 20 × 1 for the 1D convolution operation (stride = 1, padding = 0); next, for the 1D pooling operation, the size was 2 × 1. After convolution layer, two gated recurrent units were applied with 40 nodes in each layer. Finally, we used a fully connected multilayer perceptron with softmax activation for the final discrimination of sleep disorders.

### 2.4. Implementation

To implement the constructed SDN model, the software and hardware specifications are as follows. The deep learning environment consisted of the Keras [41] library with a TensorFlow backend [42] and a workstation with an Intel CPU (i9-9900X @3.5GHz) and NVIDIA GPU (GeForce RTX 3080) for the SDN. Data processing of the ECG was performed using MATLAB (R2020b ver.).

### 2.5. Evaluation Index

The constructed SDN uses the F-measure to evaluate the correct classification according to class equality. We can obtain the F-measure by combining two evaluation measures, precision and recall. These are defined as follows:

precision = TP/(TP + FP)(4)

recall = TP/(TP + FN)(5)

where TP, FP, and FN denote true positives, false positives, and false negatives, respectively; they represent the numbers of the respective events.

The F1 score, better known as the unbalanced data set, is computed based on the sample proportion of precision and recall and is given by

F1 = 2 × (precision × recall)/precision + recall(6)

## 3. Results

The proposed SDN can be utilized to achieve multiclass classification for four different sleep disorders, namely INS, PLM, RBD, and NFE, and the CNT group. The main contribution of this study is that it shows the possibility of sleep disorders classification based on ECG signal and represents the ECG feature maps that are automatically extracted by the proposed SDN model. Furthermore, we have noticed that ECG feature maps are shaped or contained the specific patterns for each sleep disorder.

The results of the proposed SDN model based on a single-lead ECG signal are presented in Table 4. The performance of the proposed SDN model was evaluated using the evaluation matrix with precision, recall, and F1 score. We obtained robust performances for the automatic classification of multiclass sleep disorders using a single-lead ECG signal without any hand-crafted features. The results of the test set present F1 scores of 99.0%, 97.0%, 97.0%, 95.0%, and 98.0% for the CNT, INS, PLM, RBD, and NFE groups, respectively.

**Figure 2:**
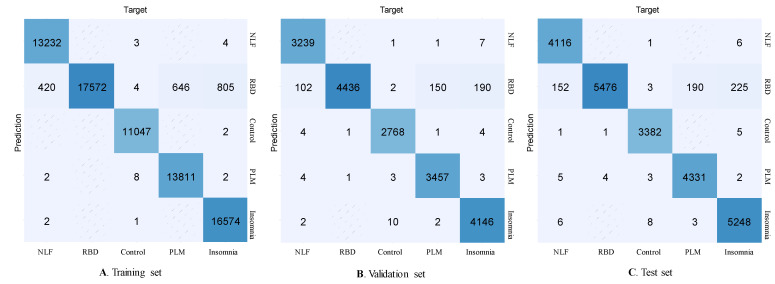
Confusion matrix of the proposed SDN model for the automatic classification of sleep disorders based on a single-lead ECG signal. The performances of the training set (**A**), validation set (**B**), and test set (**C**), respectively.

The confusion matrix of the proposed SDN model for the automatic classification of sleep disorders based on deep learning is presented in Figure 2. The sleep disorder events are equally distributed in each dataset (the training, validation, and test sets).

## 4. Discussion

**Figure 3:**
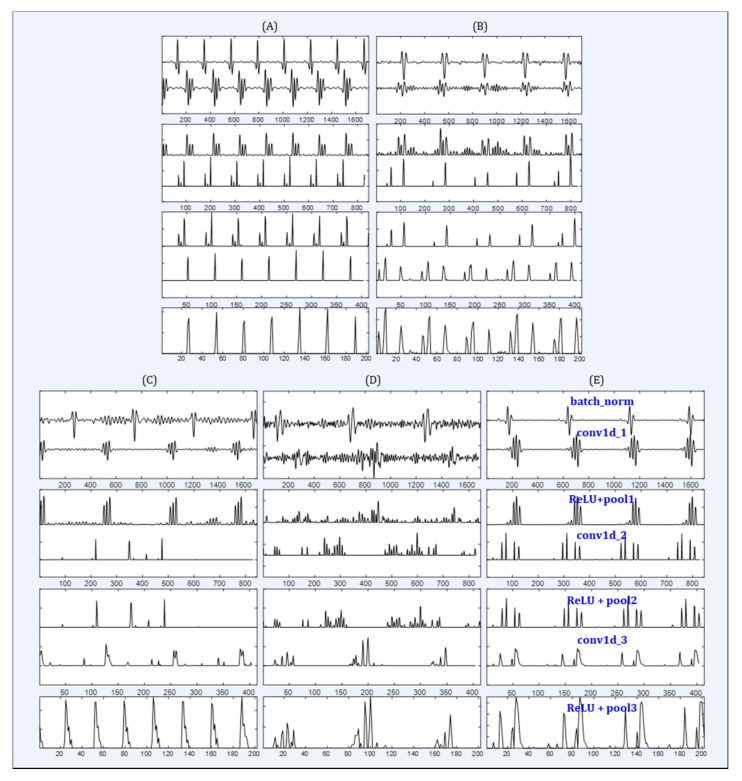
Example of the intermediate feature map of the designed deep learning SDN model for automatic classification of sleep disorders using a single-lead ECG signal. Intermediate feature maps of (**A**) INS, (**B**) PLM, (**C**) CNT, (**D**) RBD, and (**E**) NFE groups.

In this study, we analyzed the single-lead ECG signals of subjects with four important sleep disorders such as INS, PLM, RBD, and NLF through the proposed SDN model. To the best of our knowledge, there were no similar previous studies in which the single-lead ECG signals were analyzed in accordance with the patients who suffered from sleep disorders. Therefore, it is hard to compare the performances with the previous studies that used ECG signals for the classification of sleep staging and detection of sleep apnea, due to the different outcomes. The proposed SDN model was used as not only automatic classifier for sleep disorders, but also an automatic feature extractor from the input single-lead ECG signal at each layer. We illustrated the intermediate processing phase of the proposed SDN model for each subject group in Figure 3. As a result, we can deduce the difference between outputs in the subject groups and in each layer, such as convolutional, activation, and pooling. It is difficult to find a certain pattern for each group, but we can notice some morphological differences in each group. The irregularity pattern was demonstrated in the ECG signal of subjects with CAP diseases as RBD (Figure 3D), NFE (Figure 3E), and with the control group (Figure 3C). It means that ECG-based classification for the discriminating sleep disorders is a very hard task. However, the proposed SDN model can classifying the type of sleep disorders with outstanding performances using only a single-lead ECG signal.

The single-lead ECG signals have been used in various studies to detect or screen certain sleep disorders based on signal processing and machine-learning techniques. Most of these studies have focused on sleep stages and quality [21,22,23,24,25,26,27], followed by sleep-disordered breathing (e.g., sleep apnea and hypopnea events) [19,20]. In studies related to sleep stage classification and sleep quality based on electrocardiogram, Adnan et al. proposed a method of predicting sleep efficiency through sleep–wake classification from a single-lead ECG signal. Xiao et al. classified sleep stages based on a random forest using the heart rate variability from the ECG signal. In addition, Yucelbas et al. studied a method of classifying sleep stages by applying the morphological features of a single-lead ECG signal to several machine learning algorithms such as SVM, variational mode decomposition, Hilbert Huang transform, and morphological method.

Wei et al. proposed a new method for classifying sleep stages by applying ECG signals to a deep neural network model to determine the sleep stages. For a study on the ECG-based detection of sleep apnea, Mendez et al. [17] proposed a method based on an autoregressive model for automatic screening of obstructive sleep apnea from a single-lead ECG signal, and Chen et al. [18] proposed an automated algorithm for sleep apnea screening. Since the effects of sleep staging and sleep apnea related to ECG signals were proved clinically, many studies had presented a variety of methods for detection or classification of sleep apnea based on single-lead ECG signal. However, to the best of our knowledge, there are few studies on the automatic screening or detection of sleep disorders including INS, PLM, RBD, and NFE using an ECG signal. The proposed deep learning-based SDN model is a potentially alternative method for automatic screening or detecting sleep disorders conveniently and accurately. Since, the proposed SDN model has some advantages over previous studies in terms of technology and cost. At first, the SDN model is consisted of CNN and RNN architecture to consider the morphological and sequential characteristics of ECG signal. This combined architecture may lead to robust performances for the detection of sleep disorders. Finally, the SDN model with the simple architecture is expected to reduce computational and economic costs.

There are some limitations of this study. Firstly, the dataset that the CAP sleep dataset was derived from is a small population; a further study is required to cover larger and diverse datasets. Secondly, we used only a single-lead ECG signal extracted from the PSG recordings that were measured during sleep; more physiological signals, such as photoplethysmogram, EEG, and EMG, which are suitable for healthcare measurement, should be considered in future studies. Thirdly, we used nocturnal ECG when employing PSG in this study; however, the study should be continued for the ECG signal measured during general activity in daily life. Finally, we used a very simple and well-known CNN model to design the SDN model for the automatic classification of sleep disorders; recent and advanced deep learning models, such as an attention network and a reinforcement-learning method, should be applied and compared in further studies.

## 5. Conclusions

The proposed AI-enabled SDN model can be applied in digital healthcare services and used for the screening and monitoring of sleep disorders in home and hospitals. In addition, this study can be an experimental study on the automatic classification of sleep disorders using a single-lead ECG signal based on deep learning. The proposed SDN model can classify subjects with or without sleep disorders using a single-lead ECG signal. It can extract the sleep disorder features and patterns from the morphology of the input ECG signal. We attempted to explain how sleep disorders affect the morphology of ECG signals. The SDN delivered a very high-performance level with F1 scores of 99.0%, 97.0%, 97.0%, 95.0%, and 98.0% for the CNT, INS, PLM, RBD, and NFE groups, respectively. Therefore, the proposed SDN model can be an appropriate and simple tool for sleep disorders detection, sleep monitoring, and screening. Further, a validation study should be conducted in the future for the SDN that covers larger and more diverse subject groups.

## Figures and Tables

**Table 1:** Demographics of the study population.

Groups	Number (*N*)	Sex (M:F)	Age (Mean ± Std.)
CNT	7	3:4	32.6 ± 3.7
INS	7	2:5	58.9 ± 10.2
PLM	7	5:2	54.7 ± 7.0
RBD	7	6:1	72.3 ± 6.7
NFE	7	3:4	26.3 ± 8.0
Total	35	19:16	48.9 ± 2.1

Note: CNT—control, INS—insomnia, PLM—periodic leg movement, RBD—REM sleep behavior disorder, NFE—nocturnal frontal lobe epilepsy.

**Table 2:** ECG datasets for the training, validation, and test sets in this study.

Groups	Training Set	Validation Set	Test Set	Total
CNT	11,087	2713	3444	17,244
INS	17,475	4309	5439	27,223
PLM	14,439	3675	4478	22,592
RBD	17,490	4458	5543	27,491
NFE	13,644	3379	4264	21,287
Total	74,135	18,534	23,168	115,837

Note: CNT—control, INS—insomnia, PLM—periodic leg movement, RBD—REM sleep behavior disorder, NFE—nocturnal frontal lobe epilepsy.

**Table 3:** Detailed structure of the proposed SDN algorithm.

No.	Layers	Filters, Kernel Size	Output Shape	Parameters
1	batchnorm_1	=	5120 × 1	4
2	conv1d_1 maxpool1d_1 dropout_1	100, 50 × 1 2 × 1 *p* = 0.25	5071 × 100 2535 × 100	5100
3	conv1d_2 maxpool1d_2 dropout_2	80, 40 × 1 2 × 1 *p* = 0.25	2496 × 80 1248 × 80	320,080
4	conv1d_3 maxpool1d_3 dropout_3	60, 30 × 1 2 × 1 *p* = 0.25	1219 × 60 609 × 60	144,060
5	conv1d_4 maxpool1d_4 droput_4	40, 20 × 1 2 × 1 *p* = 0.25	590 × 40 295 × 40	48,040
6	gru_1 droput_5	40 *p* = 0.25	295 × 40	9840
7	gru_2 droput_6	40 *p* = 0.25	295 × 40	9840
8	dense_1	5	40	205
Total	4 conv1d layers (280 filters), 2 gru (80 units)			537,165

**Table 4:** Performance of the proposed SDN model for the automatic classification of sleep disorders based on a single-lead ECG signal.

Groups	Precision			Recall			F1-Score		
	Train	Valid	Test	Train	Valid	Test	Train	Valid	Test
CNT	1.00	1.00	0.99	1.00	0.99	0.99	1.00	1.00	0.99
INS	1.00	1.00	0.99	0.95	0.95	0.95	0.98	0.97	0.97
PLM	1.00	1.00	0.99	0.96	0.96	0.95	0.98	0.98	0.97
RBD	0.90	0.91	0.91	1.00	1.00	1.00	0.95	0.95	0.95
NLF	1.00	1.00	1.00	0.97	0.97	0.96	0.98	0.98	0.98

## Data Availability

The data presented in this study are available on request from the corresponding author.
